# A detailed map of coupled circadian clock and cell cycle with qualitative dynamics validation

**DOI:** 10.1186/s12859-021-04158-9

**Published:** 2021-05-11

**Authors:** Adrien Rougny, Loïc Paulevé, Michèle Teboul, Franck Delaunay

**Affiliations:** 1 Biotechnology Research Institute for Drug Discovery, National Institute of Advanced Industrial Science and Technology (AIST), Aomi, Tokyo Japan; 2 Computational Bio Big Data Open Innovation Laboratory (CBBD-OIL), AIST, Aomi, Tokyo Japan; 3 Bordeaux INP, CNRS, LaBRI, UMR5800, Univ. Bordeaux, 33400 Talence, France; 4 CNRS, INSERM, iBV, Université Côte d’Azur, Nice, France

**Keywords:** Biological oscillators, Cell cycle checkpoints, Reaction maps, Model checking, Boolean modelling, Control, SBGN

## Abstract

**Background:**

The temporal coordination of biological processes by the circadian clock is an important mechanism, and its disruption has negative health outcomes, including cancer. Experimental and theoretical evidence suggests that the oscillators driving the circadian clock and the cell cycle are coupled through phase locking.

**Results:**

We present a detailed and documented map of known mechanisms related to the regulation of the circadian clock, and its coupling with an existing cell cycle map which includes main interactions of the mammalian cell cycle. The coherence of the merged map has been validated with a qualitative dynamics analysis. We verified that the coupled circadian clock and cell cycle maps reproduce the observed sequence of phase markers. Moreover, we predicted mutations that contribute to regulating checkpoints of the two oscillators.

**Conclusions:**

Our approach underlined the potential key role of the core clock protein NR1D1 in regulating cell cycle progression. We predicted that its activity influences negatively the progression of the cell cycle from phase G2 to M. This is consistent with the earlier experimental finding that pharmacological activation of NR1D1 inhibits tumour cell proliferation and shows that our approach can identify biologically relevant species in the context of large and complex networks.

**Supplementary Information:**

The online version contains supplementary material available at 10.1186/s12859-021-04158-9.

## Background

The circadian clock is an adaptive mechanism that allows most organisms to anticipate the daily variations in their environment resulting from the rotation of the earth. In mammals, this system is present in virtually all cells and consists of a complex genetic network including interconnected transcriptional/translational feedback loops and that oscillates with a period close to 24 h [1]. This mechanism interacts with many other pathways to temporally coordinate basic cellular functions such as signalling, metabolism, transport, autophagy and the cell cycle. Although circadian rhythms of cell division have been observed for nearly a century, it is only recently that molecular mechanisms connecting the circadian clock and the cell cycle mechanisms have been uncovered [2–4]. Importantly, the dynamics of the two systems were shown to be robustly coupled in dividing mammalian cells, suggesting a bidirectional interaction [5, 6]. Understanding the coupling between the cell cycle and the circadian clock oscillators in healthy and tumor cells is of primary conceptual and biomedical significance. Indeed it is unknown how cells integrate multiple oscillators operating simultaneously. Further, accumulating evidence supports the notion that circadian disruption is a hallmark of cancer cells in addition to deregulated cell cycle, apoptosis and DNA repair pathways, avoiding immune destruction, metabolic reprogramming, etc. [7]. A better understanding of these links may also pave the way to the development of timed chemotherapy [8].

Given the complexity of the cell cycle machinery, addressing experimentally this issue is particularly challenging, this being mainly due to the fact that perturbation experiments are not specific and often difficult to interpret as a result of numerous, complex, or even artefactual interactions. Mathematical modelling has become instrumental as a complementary approach to the classical genetics and biochemical analysis of complex dynamical biological systems such as the circadian clock and the cell cycle [9]. In contrast to classical continuous or stochastic modelling frameworks which remain limited to a relatively low number of variables, qualitative and logical models are able to accommodate large networks [10, 11] and provide robust predictions [12].

Molecular interaction maps have long been used to describe the precise molecular mechanisms underlying biological systems graphically. Such representations have benefited from the development of standards, notably the Systems Biology Graphical Notation (SBGN) [13], as well as of a number of editing or visualization tools [14]. Numerous large scale maps are now made readily available in databases or repositories [15–18], and have become a fundamental resource in Systems Medicine [18]. Such maps, by nature, represent static knowledge of molecular mechanisms. However, they can be used as a backbone for the construction of various kinds of mathematical models [19]. A number of graphical tools allow users to build dynamical models together with their underlying molecular networks, represented as maps [20]. Moreover, methods and tools have been proposed to generate dynamical models automatically from maps, using quantitative [21–23] or qualitative [23–25] interpretations. Such methods are particularly suited to build large scale Boolean models, since their parameterization might depend solely on the structure of the map.

In this paper, we introduce a detailed SBGN Process Description (SBGN PD) [26] map of the molecular interactions regulating the circadian clock, curated from 190 references. It aims at providing an annotated summary of the state-of-the-art knowledge of the molecular mechanisms underlying this biological system. Additionally, our map was built so as to include a number of species connecting the circadian clock to the mammalian cell cycle and that play a key role in their coupling. In order to validate the content of our map in the context of this coupling, we merged it with the detailed map of the mammalian cell cycle (RB/E2F pathway) provided in [27], and generated a qualitative dynamical model from the resulting map. We subsequently checked whether our model could reproduce essential known behaviors of each cycle and of their coupling, and made some predictions of regulations of the cell cycle progression by proteins of the circadian clock.

## Results

### The circadian clock map

**Fig. 1:**
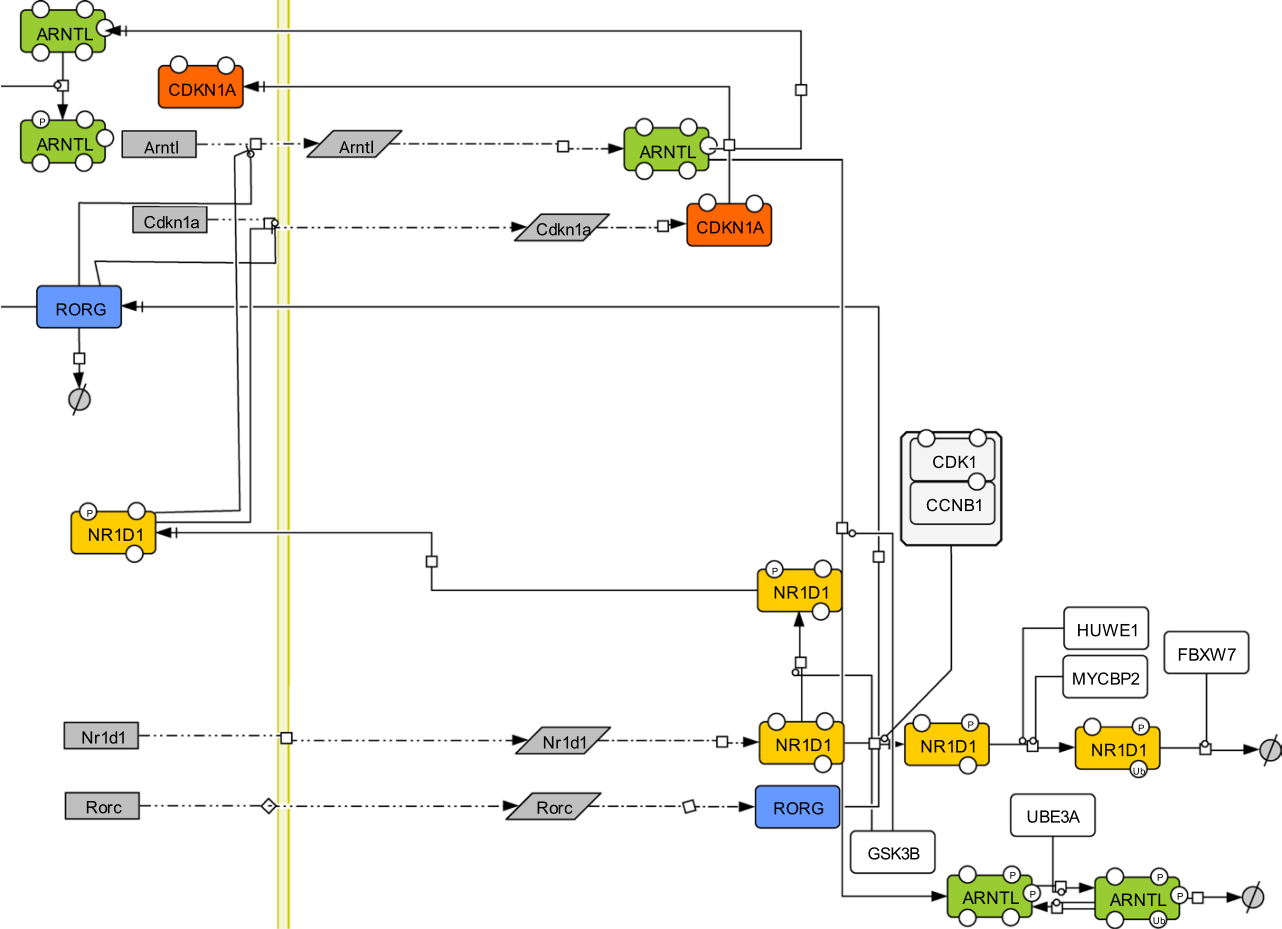
Detailed view of the NR1D1 subnetwork, visualized in CellDesigner. The subnetwork is represented using the CellDesigner format [33], which is compatible with the SBGN Process Description language [26]

Several maps of the circadian clock mechanism have been generated and made publicly available through the PANTHER (http://www.pantherdb.org/) [28], Reactome (https://reactome.org/) [15] or KEGG (https://www.genome.jp/kegg/) [18] databases. Although these interactive maps are useful tools for data exploration and pathway analysis, they all suffer from specific limitations ranging from absence or over abstraction of key regulatory modules to unbalanced complexity, to format conversion and layout rendering issues. This makes their direct use as sub-networks of larger maps time consuming if not virtually impossible. We therefore undertook the construction of a novel map of the mammalian core circadian clock devoid of such limits with two main objectives in mind: (1) the map should be able to capture the known dynamics of the circadian clock when analyzed using the qualitative modelling approach used in this paper and (2) allow the merging with an existing SBGN PD map recapitulating the cell cycle network [27]. The SBGN PD circadian clock map reported here displays key experimentally validated nodes and their interactions known to play a significant role in the dynamics of the core circadian clock present in mammalian peripheral cells [1, 29]. An excerpt of this map is given in Fig. 1, while the full map is given in Additional file 2: Figure S1. In brief, the core negative feedback loop of the clock mechanism involves the transcriptional activation of the *Per1, Per2, Cry1 and Cry2* genes by the CLOCK:ARNTL heterodimer. Upon translation, the PER1, PER2 CRY1 and CRY2 proteins undergo various posttranslational modifications, form a complex and ultimately translocate to the nucleus where they inhibit the CLOCK:ARNTL activity. This loop is interwoven with a another important loop generated by the NR1D1 nuclear receptor which represses the transcription of *Arntl* while its gene is a transcriptional target of the CLOCK:ARNTL heterodimer. The map contains 156 species associated with 14 genes, 14 mRNA and 43 proteins and their prosttranslational modifications, association or degradation, 4 simple molecules and heat. One hundred and eighteen processes include 21 associations, 7 dissociations, 14 transcriptional and 14 translational events, 42 posttranslational modifications and 20 transports occurring between the cytoplasm and either the nucleus or extracellular compartments. Heat and glucocorticoids (GC) displayed in the extracellular space represent important inputs contributing to the synchronization of peripheral clocks [30, 31]. The circuitry shown in this map was assembled from several biologically meaningful modules. For instance, the CLOCK:ARNTL heterodimer and its associated posttranslational regulation by PARP1, CSNK2A, GSK3B, and SIRT1 forms the core of the nuclear module controlling the transactivation of eight different targets (*Nr1d1*, *Rorc*, *Cry1*, *Cry2*, *Per1*, *Per2*, *Wee1*). As NR1D1 undergoes extensive posttranslational regulation by GSK3B, HUWE1, MYCBP2, and FBXW7, which is potentially important in the context of the coupling with the cell cycle, this was detailed in another module. The PER and CRY proteins form the core of both a cytoplasmic module that describes their association and posttranslational regulation by kinases (CSNK1D, CSNK1E), ubiquitin ligases ($\beta$TRCP, FBXL3) deubiquitinases (USP2, HAUSP) and a nuclear module recapitulating the mechanism through which they inhibit the transcriptional activity of the CLOCK:ARNTL heterodimer. This module is connected to the TP53 nuclear module through PER2. Finally, a module recapitulating the action of HSF1 and glucocorticoid signaling on *Per2* gene expression was included as synchronizing inputs. The CDK1, CHEK2:ATM, CDKN1A, CDKN2a, TP53 and WEE1 proteins or complexes present within the cell cycle map and connected to the clock network through physical interaction or gene regulation have been included in the circadian clock map. These proteins act at various phases of the cell cycle and the directionality of the influence is primarily from the clock to the cell cycle with only two interactions out of eight in the opposite direction (Table 1). Although not present in the cell cycle map the MYC protein and its inhibitor MIZ1 have been included as this link may be relevant to the cancer cell context [32]. Upon visualization in CellDesigner, non experts can easily navigate this map which is further augmented by nearly 300 links to external databases in the present version.

**Table 1:** Summary of experimentally validated molecular interactions between the circadian clock and the mammalian cell cycle molecular networks

Clock		Cell cycle	Phase	Mechanism	Output	Ref.
ARNTL:CLOCK	$\rightarrow$	*Wee1*	G2/M	Transcriptional	Activation	[2]
NR1D1	$\rightarrow$	*Cdkn1a*	G1	Transcriptional	Repression	[4]
RORG	$\rightarrow$	*Cdkn1a*	G1	Transcriptional	Activation	[4]
NONO	$\rightarrow$	*Cdkn2a*	G1	Transcriptional	Activation	[34]
NR1D1	$\leftarrow$	CDK1	G2/M	Phosphorylation	Degradation	[35]
PER1	$\rightarrow$	CHEK2:ATM	S	Complex formation	DNA damage induced apoptosis	[3]
CRY2:TIM	$\rightarrow$	CHEK2:ATM	S	Complex formation	Defective intra-S checkpoint	[36]
PER2	$\leftarrow$	TP53	S	Nuclear translocation	Stabilisation	[37]

Arrows represent the direction of the influences ($\rightarrow$: from the circadian clock to cell cycle; $\leftarrow$: from the cell cycle to the circadian clock). All reported interactions have evidence in the literature

### Merging of the circadian clock and cell cycle maps

For the validation of our map and its coupling with the cell cycle, we merged it with the detailed map of the mammalian cell cycle (RB/E2F pathway) provided in [27]. Merging the two maps first required a harmonization step, which was realized programmatically. This step was necessary since our map was built so it could also be used as a standalone, and followed slightly different or updated nomenclatures compared to the cell cycle map. Moreover, some entities were not described with the same level of details in the two maps, e.g., with respect to phosphorylation sites. Based on our knowledge of the maps, we manually listed these entities, and the required modifications. Rather than proceeding to these modifications manually, we implemented them in a program using the *csbgnpy* Python library, which can modify the conceptual models underlying SBGN PD maps (see “Methods” section for more details). Such a programmatic approach has several advantages: the original maps can be used as inputs, the list of modifications is made explicit, and the merging can be repeated on updated versions of the input maps. All changes made to both maps are described in details in Additional file 1. The resulting merged map contained 348 species and 285 processes.

### Validation using qualitative dynamical analysis

We checked whether the merged map could reproduce essential known behaviors of each cycle and of their coupling. Aiming at introducing as few *a priori* as possible, notably related to kinetic parameters and molecular concentrations, our dynamical model was generated from the map using a qualitative interpretation. In this interpretation, each entity of the map is either “absent” or “present”, and its state may change according to the processes specified in the map.

**Fig. 2:**
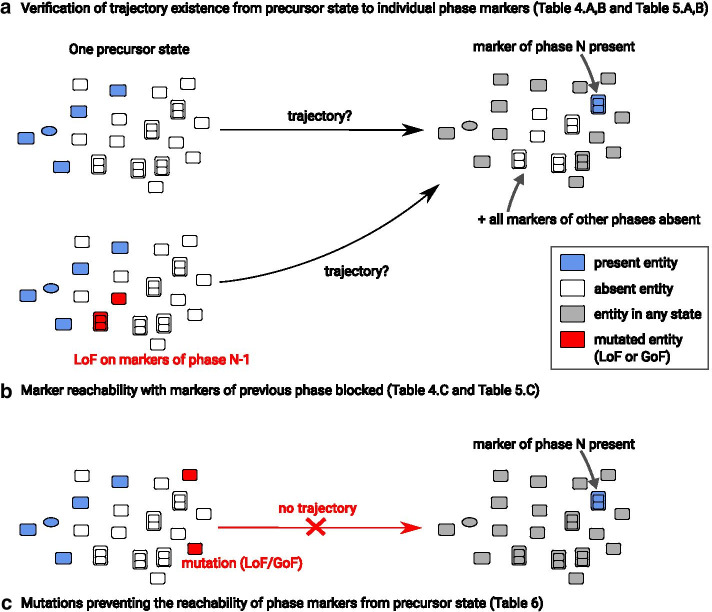
Illustration of the qualitative validations and predictions performed on the dynamical model. A precursor state is composed of the least number of entities to be “present” (represented in blue) so that all processes of the map can be “switched on” in the future. **a** For a given phase N, we check for the existence of trajectories from a precursor state leading to a state where at least one marker of that phase phase is “present” and all markers of all other phases are “absent”. **b** Same as in **a**, but using a model where all markers of the phase directly preceding phase N (i.e., of phase N − 1) are disabled by a Loss-of-Function (LoF). **c** For a given phase N and a given marker of that phase, we compute the mutations [Loss-of-Function (LoF) or Gain-of-Function (GoF)] that prevent the existence of trajectories leading to a state where this marker is “present”

Specifically, we checked whether our model could reproduce a correct progression of both cycles, and predict correct influences of entities of one cycle on the progression of the other. The progression of each cycle is characterized by a (repeated) succession of phases. Hence, for each cycle, we checked whether our model could reproduce a correct succession of these phases. To achieve that, we considered a number of markers for each phase of both cycles, that are entities specifically present in that phase, and checked whether they could be reached from specific initial states in different settings (see Fig. 2). The cell cycles exhibits four well-known phases (G1, S, G2, and M), for which markers had previously been defined [24]. We considered these markers in this study, and likewise divided phases G1 and S into two early and late sub-phases as those phases show measurable changes between their beginning and end (Table 2). As for the circadian clock, we identified four different phases corresponding to activity peaks of specific core clock entities (that we subsequently name RORG, SIRT1, ARNTL-CLOCK and PER-CRY) [38], that we considered as markers for these phases (Table 3).

**Table 2:** Markers of the phases of the cell cycle

Phase	Marker	Compartment
early G1	CCND1:CDK6(P@Thr):CDKN1B	Nucleoplasm
	pRB:E2F1:DP1:SWI:SNF:HDAC1	Nucleoplasm
	SWI:SNF:HDAC1:SUV39H1:pRB:E2F1:DP1	Nucleoplasm
	CCND1:CDK4(P@Thr172):CDKN1B	Nucleoplasm
	pRB:E2F1:DP1:SWI:SNF:HDAC1:SUV39H1:HP1gamma	Nucleoplasm
late G1	pRB(P):E2F1:DP1:SWI:SNF:HDAC1	Nucleoplasm
	pRB(P|P):E2F1:DP1:SWI:SNF	Nucleoplasm
early S	CCNE1:CDK2(P@Thr160)	Nucleoplasm
late S	CCNA2:CDK2(P@Thr160)	Nucleoplasm
G2	CCNB1:CDK1(P@Tyr15)	Cytoplasm
	CCNB1:CDK1(P@Tyr15|P@Thr14)	Cytoplasm
	CCNB1:CDK1	Cytoplasm
	CCNB1:CDK1(P@Thr14)	Cytoplasm
M	CDC25(P)	Nucleoplasm
	WEE1(P@Ser53)	Nucleoplasm
	CCNB1:CDK1(P@Thr161)	Nucleoplasm
	CDC20(P)	Nucleoplasm

The markers for each phase of the cell cycle are given under a textual form. The names used to describe the markers are the labels of the corresponding entities in the map. Subunits in complexes are separated using the “:” character. Text in parantheses gives the post-translational modifications of a given entity (protein or complex subunit). For example, “P@Thr172” represents a phosphorylation at threonine 172. A same entity may have more than one post-translational modifications, separated in the text by the “|” character

**Table 3:** Markers of the phases of the circadian clock

Phase	Marker	Compartment
RORG	RORG	Nucleoplasm
SIRT1	SIRT1	Nucleoplasm
ARNTL-CLOCK	ARNTL(Ac@L538|P@S90):CLOCK(ADPr):CSNK2A	Nucleoplasm
PER-CRY	PER1:CRY1:CRY2:ARNTL(Ac@L538|P@S90): CLOCK(ADPr):PER2(Ac):CSNK1D:CSNK1E	Nucleoplasm

The markers for each phase of the cell cycle are given under a textual form. The names used to describe the markers are the labels of the corresponding entities in the map. Subunits in complexes are separated using the “:” character. Text in parantheses gives the post-translational modifications of a given entity (protein or complex subunit). For example, “P@Thr172” represents a phosphorylation at threonine 172. A same entity may have more than one post-translational modifications, separated in the text by the “|” character

#### Construction of the dynamical model

We built the dynamical model from the qualitative dynamical interpretation introduced in [24] (see “Methods” section). In this interpretation, each entity and process of the map is considered as a binary variable whose state (“absent”/“present” for entities, “switched off”/“switched on” for processes) can evolve in time, subject to the state of all entities and processes of the map. For instance, a process can be “switched on” whenever all its reactants are “present” and its modulation logic is satisfied (stimulator “present”, or inhibitor “absent”, for example). Different strategies for computing possible state changes exist [24] and may impact substantially the predictions.

The interpretation we opted for in this study aims at introducing as few *a priori* assumptions as possible, so as not to arbitrarily exclude specific dynamical behaviors. In particular, it does not add constraints between the joined stimulatory and inhibitory activities of entities modulating a process: a process may be “switched on” if one of its stimulators is “present”, and “switched off” if one of its inhibitor is “present”. The application of rules for changing states of entities and processes is then non-deterministic. Whereas enabling many behaviors, it provides guarantees with respect to stochastic interpretations of the map, without having to specify kinetics (see “Methods” section and [39]). The resulting dynamical model is expressed as automata networks, or equivalently 1-safe Petri nets, from which one can make simulations of the evolution of the states of entities and processes of the map, and make exhaustive analysis of possible trajectories. The model obtained from the merged map resulted in 609 automata and 1220 rules for state changes.

#### Dynamical properties

From an initial state, we say a target state is *reachable* whenever there exists a sequence of rule applications (trajectory) from the former to the later state. By extension, we say that an entity is *reachable* from a given initial state if there exists a reachable state where the entity is “present”.

We consider that our model is in a particular phase of a cycle if at least one marker of that phase is “present” and all markers of all other phases of the cycle are “absent”. This enables checking whether a given phase is reachable (Fig. 2a). More generally, one can verify whether there exists a trajectory going through an arbitrary sequence of phases, possibly with repeats.

We can furthermore prevent a phase from being reachable by *disabling* all markers of that phase, where these markers are prevented from being “present”. This allows us to check whether *all* trajectories leading to a particular phase go through another phase: whenever a reachable phase Y becomes non-reachable by disabling all the markers of phase X, then all trajectories rely on at least one marker of X at some time (Fig. 2b), from the same initial state.

Finally, we studied *mutations* of one or several entities which prevent the reachability of individual phase markers (Fig. 2c). A mutation either disables an entity (making it impossible to be “present”), or forces its presence (making it impossible to be “absent”).

#### Choice of the initial state

As illustrated in Fig. 2, the validation of the model involves verifying the existence of trajectories leading to specific states from an initial state of the model, i.e. a set of entities defined as “present” at the initial time step. The definition of a suitable initial state is critical in such analyses as it has a preponderant effect on reachability results. As the exact coupling of the two cycles has still not been elucidated, it is not possible to define an initial state that would represent an actual state of the coupling that could be observed experimentally. Moreover, due to the large number of entities of the model, it is also not possible to consider all possible initial states (i.e. the power set of the set of entities of the map, all processes being “switched off” in the initial state), which is exponential with regard to the number of entities of the map. Hence, we built a small set of initial states by constraining the set of all possible initial states, mostly based on dynamical considerations. In the rest of the analysis, we call these specific initial states *precursor states*. The constraints we considered to compute these precursor states are described hereafter.

We first reduced the number of possible precursor states by considering a constraint based on *a priori* knowledge. The complex formed of the three proteins pRB (unphosphorylated), DP1, and E2F1 plays a major role in the beginning of the cell cycle: E2F1 becomes active and triggers the transition from G1 to S when it is freed from the complex it forms with pRB subsequently to the phosphorylation of the latter [27]. Hence, we constrained all precursor states to include this complex, rather than the three proteins in their free forms.

The rest of the constraints were defined so that the resulting precursor states would not arbitrarily prevent the reachability of some entities of the model. To this end, we further reduced the set of all possible precursor states by considering only those that would allow all entities of the map to be reached when not considering effects of modulators on processes. Such a constraint guarantees that any absence of reachability is due to the internal structure of the molecular network rather than to the precursor state itself. Many sets of entities satisfied this second constraint. Hence, we chose the sets of entities that both maximized the number of proteins in their most unmodified and free forms and that had the least cardinality. There were 24 such sets. Each set contained 106 entities, 102 of which were common to all sets. The four remaining entities for each set resulted from a combination of the four following alternatives. Each set contained (i) either cytoplasmic TP53 or nucleoplasmic TP53; (ii) either cytoplasmic E2F4 or nucleoplasmic E2F4; (iii) either cytoplasmic DP2 or nucleoplasmic DP2; and (iv) either NAD+, NAM, or NMN. The first three alternatives can be explained by the fact that in the merged map, TP53, E2F4, and DP2 have two possible localizations (cytoplasmic and nucleoplasmic), and that it contains cycling translocation processes from one localization to the other for each of these entities. As for the fourth alternative, it is due to the presence in the merged map of a cycle producing and consuming the three entities NAD+, NAM and NMN. All sets also contained the inputs of both maps, in particular the entities representing heat and GC in the extracellular space of the circadian clock map. This was to be expected considering the constraint used to compute the precursor states and the fact that those inputs cannot be produced in the maps. All 24 resulting precursor states were considered in the following dynamical analysis.

#### Progression of the circadian clock

We checked whether our dynamical model exhibited a correct succession of the phases of the circadian clock. As a control, we first checked for each of the 24 precursor states whether each marker of each phase was reachable from that precursor state (Table 4A). All markers of every phase were reachable for 16 of the 24 precursor states (Table 4), which all contained either of NAD+ or NMN which are involved in a key metabolic feedback loop of the clock mechanism [40]. Consistently, for the remaining eight precursor states not containing NAD+ nor NMN but NAM, no markers were reachable. This can be explained by circular necessary conditions entailed by the structure of the map: To be produced, each marker of every phase of the circadian clock requires NAD+. Yet, while NAD+ can be produced from NMN by the NMNAT enzyme that is present in every precursor state, NMN can only be produced from NAM by the NAMPT enzyme that is not in these precursor states, and whose gene necessitates the presence of the marker of phase ARNTL-CLOCK to be transcribed. As a result, no markers can be produced from these precursor states, which prevents their reachability.

For the 16 precursor states from which all markers were reachable, we subsequently checked whether each phase could be reached (Table 4B). All phases could be reached. Hence, we further checked whether our model could reproduce the cycle induced by the succession of the four phases. For this, we checked the reachability of the sequence of the phases of the circadian clock, repeated four times (i.e. the sequence (RORG, SIRT1, ARNTL-CLOCK, PER-CRY, RORG, ...) where each phase appears four times, in order). This sequence was reachable for all 16 precursor states, showing that our model could reproduce the cycle induced by the circadian clock.

Finally, we checked whether each phase could be reached when its preceding phase was disabled for the 16 aforementioned precursor states (Table 4C). We found that all phases could still be reached except for phase PER-CRY. We could expect that phase RORG would still be reachable. Indeed, the circadian clock map was designed as a succession of phases from RORG to PER-CRY, and we hypothesize that the precursor states are close to the states of the circadian clock when in phase RORG. Hence disabling phase PER-CRY would have no effect on the reachability of phase RORG. As for the fact that phase SIRT1 could still be reached when disabling phase RORG, it can be explained by the absence in the map of an influence of active RORG on the activation of protein SIRT1, which only requires the presence of NAD+. This might suggest we are lacking knowledge regarding the succession from phase RORG to phase SIRT1, and more generally on how the activation of protein SIRT1 is linked to the other processes of the circadian clock. Finally, the result for succession from phase SIRT1 to phase ARNTL-CLOCK can be explained by the processes protein SIRT1 modulates. Indeed, active SIRT1 stimulates both the degradation of proteins ARNTL and PER2, limiting their activity during phase SIRT1, and acting as an inhibitor of the circadian clock. Thus, it could be expected that disabling phase SIRT1 would not hamper the progression of the circadian clock and the reachability of phase ARNTL-CLOCK.

Overall, these results show that all phases can be reached from every precursor state containing NAD+ or NMN, and that our model can reproduce the cycle induced by the repeated succession of phases of the circadian clock for these precursor states. Furthermore, phase PER-CRY cannot be reached without its preceding phase ARNTL-CLOCK being reached beforehand, suggesting that our model can reproduce a correct succession for these two phases.

**Table 4:** Analysis of the progression of the circadian clock

Phase	Num. markers reachable/total
(A) Reachability of markers	
RORG	1/1
SIRT1	1/1
ARNTL-CLOCK	1/1
PER-CRY	1/1

Phase	Num. markers reachable/total
(B) Reachability of phases	
RORG	1/1
SIRT1	1/1
ARNTL-CLOCK	1/1
PER-CRY	1/1

Phase N (phase N − 1 disabled)	Num. markers reachable/total
(C) Reachability of phases with preceding phase disabled	
SIRT1 (RORG1)	1/1
ARNTL-CLOCK (SIRT1)	1/1
PER-CRY (ARNTL-CLOCK)	0/1
RORG (PER-CRY)	1/1

We tested whether each phase of the circadian clock could be reached from each precursor state, with or without its preceding phase disabled. Results are shown for the 16 precursor states containing either NAD+ or NMN. For the eight remaining precursor states rather containing NAM, no markers were reachable. For (B) and (C), the phase is reachable if at least one of its markers is reachable. (A) Reachability of the markers of each phase (control). (B) Reachability of each phase. (C) Reachability of each phase with its preceding phase disabled

#### Progression of the cell cycle

We conducted the same analysis to check whether our dynamical model exhibited a correct succession of the phases of the cell cycle. We used the same 24 precursor states as for the circadian clock, and the markers of the six phases described previously (early G1, late G1, early S, late S, G2, and M).

We found that all markers could be reached from all 24 precursor states, but one marker of phase M (phosphorylated WEE1), which was not reachable from the eight precursor states containing NAM (Table 5A). This suggests that the reachability of this marker is completely dependent on the presence of core entities of the circadian clock, which cannot be produced from these precursor states. Additionally, all phases were reachable from all precursor states (Table 5B). However, the repeated sequence of the six phases, in order, could not be reached, indicating that our model could not reproduce a cyclic behavior for the cell cycle. This could be explained by the fact that there exists no process in the cell cycle map that consumes the unique marker of phase late S. As a consequence, this marker cannot be consumed once it has been produced, and remains “present” once phase late S has been reached.

We further checked whether each phase was reachable when the phase directly preceding it was disabled, for each precursor state (Table 5C). We found that phase early G1 could still be reached when M phase was disabled, for every precursor state. This could be explained by the choice of the precursor states: because the original cell cycle map [27] was designed as a succession of processes occurring from phase early G1 to phase M, we hypothesize that all the precursor states may be close to the states of the cycle corresponding to phase early G1, and hence that disabling phase M would have no impact on the reachability of early G1. Furthermore, phase G2 could also still be reached when disabling phase late S. This result is similar to the one we found earlier [24], and can be explained by the fact that the original cell cycle map does not describe any influence of the complex CCNA2:CDK2, active at the end of phase S, on the activation of the complex CCNB1:CDK1 (marker of G2). Although such an influence has strong evidence in the literature and could have an important role in the succession from phase late S to phase G2, its mechanism is still unknown, and hence was not added to the model. All other phases but these two (early G1 and G2) could not be reached when disabling their preceding phase.

Overall these results indicate that all phases can be reached from each precursor state, and that all phases but phase early G1 and G2 cannot be reached without their preceding phase being reached first. These results are coherent with those obtained with a model built from the original map of the cell cycle alone [24], suggesting that the addition in the model of the processes of the circadian clock does not hamper the progression of the cell cycle.

**Table 5:** Analysis of the progression of the cell cycle

Phase	Num. markers reachable/total
(A) Reachability of markers	
early G1	5/5
late G1	2/2
early S	1/1
late S	1/1
G2	4/4
M	4/4 (3/4)

Phase	Num. markers reachable/total
(B) Reachability of phases	
early G1	5/5
late G1	2/2
early S	1/1
late S	1/1
G2	4/4
M	4/4 (3/4)

Phase N (phase N − 1 disabled)	Num. markers reachable/total
(C) Reachability of phases with preceding phase disabled	
early G1 (M)	5/5
late G1 (early G1)	0/2
early S (late G1)	0/1
late S (early S)	0/1
G2 (late S)	4/4
M (G2)	0/4

We tested whether each phase of the cell cycle could be reached from each precursor state, with or without its preceding phase disabled. Results were the same for the 24 precursor states except for one marker of phase M, which was not reachable from the eight precursor states containing NAM (shown in parantheses). For (B) and (C), the phase is reachable if at least one of its markers is reachable. (A) Reachability of the markers of each phase (control). (B) Reachability of each phase. (C) Reachability of each phase with its preceding phase disabled

#### Control of checkpoints

How mechanisms described in the circadian clock map may affect the progression of the cell cycle, and vice-versa, is key for understanding the potential intertwined control of the two cycles. We addressed this question by looking for processes controlling the reachability of the different cycle checkpoints, specified by their markers. Due to the size of the model, we used methods based on logical deduction to avoid a naive screening of all possible candidates. Moreover, we relied on formal approximations of dynamics, which result in a correct but non-exhaustive list of controls (see “Methods” section for more details).

Our analysis identified sets of simultaneous mutations, being either Loss-of-Function (LoF) or Gain-of-Function (GoF), which ensure that one marker of a checkpoint is no longer reachable from the precursor state. We report here the entities from the circadian clock map that are involved in the sets of mutations interfering with the checkpoints of the cell cycle, and similarly, the entities of cell cycle map interfering with the checkpoints of the circadian clock.

*Control of cell cycle checkpoints by entities of the circadian clock map* Starting from the early G1 phase, Table 6 lists the mutations of entities of the circadian clock map which can prevent the activation of at least one marker of a phase of the cell cycle. Blockade of the reachability of markers of phases early G1, late G1 and late S upon HSP90 LoF is likely due to the direct role of the protein in the cell cycle process. HSP90 is a chaperone protein that is required for the maturation and stability of a large number of proteins including entities of the cell cycle machinery such as CDK4 and CDC37 present in the cell cycle map [41]. Further, pharmacological inhibition of HSP90 with 17-Allylamino-Geldanamycin and its derivatives has an antitumor activity in preclinical models and cancer patients [42, 43]. LoF preventing reachability of markers of phases G2 and M includes mutations impairing directly or indirectly (CSNK2A, PARP1, MYC and MIZ1) CLOCK-ARNTL function which is the core transcription factor complex driving the circadian clock mechanism and the expression of the G2/M kinase WEE1 [2]. LoF of CLOCK-ARNTL function also leads to increased expression of the CDK4/6 inhibitor *Cdkn1a* as the NR1D1 transcriptional repressor is no longer produced [4]. The impact of WEE1 LoF on the reachability of markers of phase G2 lies in its role as the kinase that phosphorylates CDK1 at Tyr15 during G2. Intriguingly, this analysis revealed that NR1D1 GoF participates in preventing activation of one of the G2 and M markers. According to the analysis of the merged map, the activation of NR1D1 can prevent the activation of the complex PER:CRY:CSNK1:CLOCK:ARNTL, which is necessary for the activation of the CCNB1:CDK1 phase markers of G2 and M. This is in line with data showing that experimental overexpression of NR1D1 in vivo inhibits the expression of ARNTL [44]. Expectedly, LoF for CCNB1:CDK1 which is the key driver of the G2/M transition blocked the reachability of phase M markers.

**Table 6:** Identified mutations of entities of the circadian clock controlling the reachability of at least one phase marker of a cell cycle phase

Cell cycle phase	Circadian clock entity	Mutation
early G1	HSP90	LoF
late G1	HSP90	LoF
early S	*none identified*	
late S	HSP90	LoF
G2	PER:CRY:CSNK1:CLOCK:ARNTL	GoF
	MYC:MIZ1	GoF
	WEE1	LoF
	ARNTL	LoF
	CLOCK	LoF
	CSNK2A	LoF
	MIZ1	LoF
	NAD+	LoF
	PARP1	LoF
	NR1D1	GoF
	NMN*	LoF
	NMNAT*	LoF
M	*all controls of G2 and*	
	CCNB1:CDK1	LoF

Mutations either disable an entity (LoF), or force its activity (GoF) *: only in the eight precursor states containing NMN. For G2 and M: no mutations predicted for the eight precursor states containing NAM

*Control of circadian clock checkpoints by entities of the cell cycle map* The qualitative analysis identified no mutations of cell cycle entities involved in the blocking of circadian clock markers. However, a causal and exhaustive analysis of the trajectories leading to markers activation revealed that several trajectories leading to the activation of markers of phases RORG, ARNTL-CLOCK, and PER-CRY from the precursor state rely on the activity of TP53. This link is supported by an experimentally documented bidirectional crosstalk between the circadian clock and the TP53 pathway that modulates the stability of the PER2 and TP53 proteins [45, 46].

## Discussion

### Detailed map of the circadian clock

We built a new map of the circadian clock that encompasses all known key regulatory mechanisms governing the core clock, as well as key entities that also play a major role in the mammalian cell cycle. To our knowledge, this map is the most detailed and up-to-date map made publicly available as compared to the currently existing maps from Reactome (https://reactome.org), PANTHER (http://www.pantherdb.org), and KEGG (https://www.genome.jp/kegg) databases. The circadian clock map from Reactome includes a moderately detailed core clock and numerous clock outputs while the ones from PANTHER and KEGG describe a minimalist core clock with 6 and 13 nodes respectively. Compared to these two existing resources, the present map focuses on the core clock components, includes important synchronisation input pathways and nodes present in other critical cellular processes (cell cycle, metabolism), and incorporates a detailed and updated information related to numerous posttranslational modifications (phosphorylation, acetylation, poly-ADP-ribosylation, and ubiquitination). Quantitative models with a greater level of detail in the prosttranslational regulation of the clock have been produced, but combining their precision with the size of the network of our map remains a highly challenging task [47]. The map was designed using CellDesigner [33] and is compatible with the SBGN PD standard [26]. As such, it may be easily shared, edited, and updated. Its referencing and integration in map databases, such as the Atlas of Cancer Signalling Network [48], is under consideration.

### Validation by qualitative dynamical analysis

We used qualitative approaches to ensure that the merged map provides a consistent description of the molecular processes sufficient to reproduce expected basic dynamical behavior of both cycles (the circadian clock and the mammalian cell cycle) and of their coupling. The whole validation process was realized programmatically, and was repeated throughout the construction of the circadian clock map, allowing to iteratively improve it by adding missing molecular processes, such as degradation and translocation reactions.

Specifically, we verified the correct progression of both cycles by verifying the existence of trajectories leading to phase markers of each cycle, and predicted mutations to control these trajectories. We considered 24 precursor states for this analysis. Results differed drastically between two subsets of precursor states for the circadian clock, while they were almost the same for all precursor states for the cell cycle. Indeed, for the circadian clock, the eight precursor states containing NAM hampered the reachability of all phases of the cycle. This can be explained by the structure of the network, which exhibits a positive modulation cycle between NAD+ and the marker of phase ARNTL-RORG which leads to circular necessary conditions for the reachability of all phases that are impossible to satisfy.

For the circadian clock, all phases could be reached for 16 of the 24 precursor states (as discussed above). Additionally, our model could reproduce a cycling succession of phases (repeated four times) for these same precursor states, which indicates that the processes described in the map are sufficient to entail a cycling behavior of the model. Furthermore, we found that phase PER-CRY could not be reached when phase ARNTL-CLOCK was disabled, showing that our model exhibits a correct succession from phase ARNTL-CLOCK to phase PER-CRY in all trajectories where the latter is reached. This succession underlies the core negative transcriptional/postranslational feedback loop of the clock mechanism. However, our model failed to reproduce a correct succession for the rest of the phases (phase PER-CRY to phase RORG, phase RORG to phase SIRT1, and phase SIRT1 to phase ARNTL-CLOCK). The fact that phase RORG could still be reached when disabling phase PER-CRY could be due to the way the map or the precursor states are built, and could be further studied by defining initial states corresponding to phase ARNTL-CLOCK, i.e. checking the reachability of phase RORG from states corresponding to phase ARNTL-CLOCK when phase PER-CRY is disabled. Such an analysis could indeed give insights on the particular impact of the structure of the map on this specific succession, and possibly reveal a lack of necessary conditions in the model to progress from phase PER-CRY to phase RORG. However, defining initial states corresponding to specific phases is challenging, and cannot be addressed without new significant methodological developments (see “Future directions” section for more details). As for the fact that phase SIRT1 could still be reached when disabling phase RORG, it is likely due to the absence of an influence of active RORG on protein SIRT1. This reveals a lack of knowledge surrounding marker SIRT1 and the processes that come into play for its activation at the end of phase RORG, which are still not clearly understood and do not appear in the map. Finally, the result for the succession from phase SIRT1 to phase ARNTL-CLOCK can be explained by the overall inhibitory influence of active SIRT1 on the circadian clock (by inhibiting ARNTL and PER2). Since active SIRT1 inhibits the progression of the cycle toward phase ARNTL-CLOCK, passing through phase SIRT1 does not constitute a necessary condition for the cycle to progress toward phase ARNTL-CLOCK in our qualitative model. This might show that progression of the circadian clock through SIRT1 and ARNTL-CLOCK are rather governed by temporal aspects, that are difficult to take into account in a qualitative setting (see “Future directions” section for more details). Overall, these results for the succession of phases seem to be coherent with experimental results showing that the circadian clock function is disrupted upon loss of function of either ARNTL or PER1/PER2 isoforms or CRY1/CRY2 isoforms while SIRT1 or RORG loss of function do not abolish clock oscillations [49, 51, 51].

For the cell cycle, all phases could be reached from all precursor states. However, our model could not reproduce a cyclic behavior for this cycle, for all precursor states. This is at least partly due to the absence of processes consuming some markers (e.g. the unique marker of phase late S). Such a property could be further studied by adding processes to the cell cycle map (e.g. degradation processes) that would ensure that all markers of the cell cycle can be consumed. Additionally, we found that phases late G1, early S, late S and M could not be reached when their preceding phases were disabled, showing that our model exhibited a correct succession from their preceding phase to these phases in all trajectories where they could be reached. The fact that phase early G1 could still be reached when phase M was disabled could be explained by the way the map or the precursor states are built. Analogously to the succession from phase PER-CRY to phase RORG in the circadian clock, this specific succession could be further studied by defining initial states corresponding to phase G2. As for the fact that phase G2 could still be reached when phase late S was disabled, it can be explained by the lack of knowledge on the precise mechanisms leading to the activation of the complex CCNB1:CDK1 by the complex CCNA2:CDK2 at the end of phase S. We hypothesise that adding such a mechanism to the cell cycle map would allow us to build a new model that would reproduce a correct succession for these phases.

### Insights in the circadian clock and cell cycle interactions

Using the merged map, we analysed how entities and processes brought by the circadian clock map can control the progression of the cell cycle, and vice versa. The analysis relied on the identification of mutations which prevent the activation of a marker of a phase. We recovered expected regulatory mechanisms of the circadian clock to the cell cycle, and identified NR1D1 as a potential inhibitor for the progression towards phase G2. This prediction corroborates observations made in the literature showing that two agonists of NR1D1 are lethal to cancer cells and oncogene-induced senescent cells [52, 53], and calls for further investigation.

As for the analysis of the influence of the cell cycle map on the circadian clock map progression, it resulted in very few predictions. This reflects the fact that there are few known mechanistic effects from the cell cycle on the circadian clock.

### Future directions

Further analysis of the dynamics of both cycles and their coupling would necessitate studying some of their temporal properties, such as their period. These properties cannot be easily analyzed using qualitative approaches, because they overlook quantitative time. Nevertheless, inhibition processes, which can regulate the duration of phase transitions, may still be captured by causal analysis by identifying entities involved in pathways of phase progressions, e.g. with cut set analysis [54], together with processes de-activating these entities.

Furthermore, the present analysis is restricted to the study of trajectories from a set of initial states from which all the entities of the map may be reached (the precursor states). Being able to delineate network states corresponding to different cycle phases would allow us to provide further insight in key entities and processes regulating the circadian clock and cell cycle progression, and their synchronization. Such states could be built *a priori*, by constraining, for each phase of each cycle, a marker of that phase to be “present” and all markers of the other phases to be “absent”. This way, however, most entities of the map would not be constrained to a particular state (“present” or “absent”), which would result in defining an exponential number of states with respect to the number of entities of the map. Such a number of states would not be tractable considering the size of the maps. Moreover, we do not know *a priori* how phases of the cell cycle and the circadian clock synchronize. Hence we would have to arbitrarily choose pairs of phase states of both cycles to build global states of the coupling, or consider all possible pairs, which would further increase the number of possible states.

Another more practical solution would be to use simulation to obtain such states experimentally. Our dynamical model could indeed be simulated from an initial state until a specific state of a phase is reached, i.e. a state where a marker of a phase is “present” and all markers of all other phases are “absent”. This method could be used to sample collection of states for each phase (provided that such states are indeed reachable), which could then be analyzed to possibly give new insights on the synchronicity of both cycles, or used as initial states for further dynamical analyses.

In future work, we also plan to study alternative dynamical interpretations of SBGN PD maps, such as CasQ [25]. This interpretation results in a model expressed in the classical Boolean network framework, where tools such as Most Permissive Boolean Networks [11] can address the dynamical analysis of networks of the scale of the merged map. It would notably help providing a finer and complete analysis of trajectories and long-term behaviors (attractors) with and without mutations, enabling states prediction at phase lockings. Indeed, our current analysis is only focused on the existence of trajectories, and cannot address the computations of attractors, due to the scale of the model with the asynchronous interpretation of reactions.

## Conclusions

In this study, we introduce a new map of the circadian clock, compatible with the SBGN standard. Key features of this map include an unprecedented level of detail, upgrading and merging capabilities and, direct use for dynamic modelling. We programmatically merge this map with the previously published map of the mammalian cell cycle, and automatically build a qualitative dynamical model from the merged map we obtain. We then validate this model by showing it can reproduce the progression through their main phases of both cycles. We subsequently use our model to make predictions on mutations that would influence the coupling of the two cycles, and notably predict that NR1D1 should inhibit the progression of the cell cycle towards phase G2. This result is corroborated by previously reported observations following which pharmacological activation of NR1D1 inhibits tumour cell proliferation, and should be investigated experimentally later. Furthermore, our study relies on generic tools for the merging and the dynamical analysis that are independent of the specific maps introduced, and could be repeated on updated versions of the cell cycle and circadian clock maps, or on maps related to completely different biological processes.

## Methods

### SBGN PD standard for maps

The Systems Biology Graphical Notation (SBGN) [13] is a standard for the graphical representation of molecular networks. It includes three orthogonal languages: the Process Description (PD) [26], used to represent reaction networks; the Activity Flow [55], used to represent influence graphs; and the Entity Relationship [56], used to represent sets of rules and relationships. We focus hereafter on the SBGN PD language, as we are interested in representing and modelling reaction networks.

SBGN PD is used to represent the precise biomolecular mechanisms underlying biological processes. As its name suggests, the central concept it permits representing is the process, that transforms pools of entities into other pools of entities, and that may be modulated by yet other pools of entities. PD defines a number of glyphs (nodes and arcs), each having a specific shape and representing a different concept. The main glyphs of the PD language are the following:

- *Entity Pool Node (EPN)*: represents a pool of entities, that is a set of identical molecules. There are 13 types of EPNs, among which the macromolecule, simple chemical, complex and nucleic acid feature.
- *Compartment Node*: represents a compartment, where entity pools are localized.
- *Process Node*: represents a process that transforms one or more entity pools into other entity pools. The reactants and products of the process are linked to it using consumption arcs and production arcs, respectively. There are six types of processes: the generic process, association, dissociation, phenotype, omitted process and uncertain process.
- *Modulation arc*: represents the modulation of a process by an entity pool. There are five types of modulation arcs: the generic modulation, stimulation, inhibition, necessary stimulation, and catalysis.
- *Logical Operator Node*: represents complex logical functions that model modulations involving more than one EPN. There are three types of logical operator nodes: the AND operator, OR operator, and NOT operator.

An actual drawing in SBGN of a network is called an SBGN map. SBGN maps may be stored and exchanged under the SBGN-ML format [57]. Multiple software allow users to draw such maps [58], one of which is CellDesigner [33]. This particular software focuses on the edition of SBGN PD maps and has been used to draw large comprehensive maps [27, 28, 48, 59, 60]. It only partially supports the SBGN-ML format and mostly relies on its own format to import and export maps. This format is an extension of the SBML format, and can be converted to SBGN-ML using the *cd2sbgnml* software [61].

### Construction of the circadian clock map

The biological knowledge providing a comprehensive understanding of the molecular circuitry governing the circadian clock (see [1] and [29] for reviews) was used to design a prototypical mammalian peripheral circadian clock map in CellDesigner 4.4.2 (http://www.celldesigner.org) using the SGBN standard (https://www.sbgn.org). The 190 peer-reviewed articles supporting this knowledge and published by December 2018 can be directly accessed from the map. This map did not intend to exhaustively describe all the known species and interactions but instead incorporate essential nodes and edges of the core clock mechanism as well as critical inputs and outputs connecting the core clock and the cell cycle machinery. Accordingly, paralogs were not included in most instances and specific accessory loops (DBP, DEC1) were also omitted in this version. As eukaryotic circadian clocks are based on interwoven transcriptional/translational feedback loops, the circadian clock map incorporates genes, mRNA and protein products with their associated posttranslational modifications (phosphorylation, ubiquitination, acetylation). However, when transcriptional regulation was not essential or not documented, only proteins were considered for some modules. It also incorporates input pathways known to be important for the synchronization of peripheral clocks such as glucocorticoid signaling and temperature. Genetic interactions not supported by mechanistic evidence have not been included. This map compiles only experimentally validated results obtained mostly in the mouse animal model. The map is enriched with more than 300 external links to the Entrez, UniprotKB, ChEBI and PUBMED databases.

### Modification and merging of the cell cycle and circadian clock maps

In order to modify and merge the cell cycle and circadian clock maps, we developed *csbgnpy*, a Python library that eases the manipulation of SBGN PD maps, at the conceptual level. With *csbgnpy*, maps can be modified programmatically without alteration of the input files, and the applied modifications can be easily tracked. *csbgnpy* extracts all the biological and biochemical concepts represented by a map (e.g. entity pools, processes, modulations) and stores them in memory under the form of Python objects that can be manipulated using simple Python code. The operations facilitated by *csbgnpy* include the creation of new concepts and their addition to a map; the search for concepts in a map; the deletion or modification of concepts of a map; and the construction of new maps by merging or intersecting two or more maps. Concepts in *csbgnpy* may also be manipulated using a new textual representation called sbgntxt, that eases their construction or identification. *csbgnpy* can read maps from different file formats such as SBGN-ML, CellDesigner and BioPAX (using third-party libraries or converters, in particular *cd2sbgnml* [61] for the CellDesigner format) and write maps to the SBGN-ML format (with no layout). *csbgnpy* is freely available at https://github.com/Adrienrougny/csbgnpy, and can be modified and redistributed under the terms of the GPLv3 license.

The modification and merging of the two maps, as well as the export of the resulting map to an SBGN-ML file, were performed programmatically using *csbgnpy*, directly from the input CellDesigner files. All changes made to both maps are described in details in Additional file 1.

### Dynamical model

#### Construction of the dynamical model

We built a qualitative dynamical model from the merged map using software *Pint* [10]. *Pint* is a static analyzer for dynamics of asynchronous automate networks (AN). It can also be used to generate a dynamical model encoded under the form of an AN from an SBGN PD map stored under the SBGN-ML format. Generation of the model relies on the *general semantics*, which is a qualitative dynamical interpretation of SBGN PD maps described in [24]. We briefly describe this interpretation and the encoding under the form of an AN hereafter.

The general semantics is a qualitative Boolean interpretation that extends the one of BIOCHAM [62]. Under this interpretation, (i) each EPN may be in one of two states “present” or “absent”; (ii) each process may be in one of two states “switched on” or “switched off”; (iii) each modulation may be in one of two states “active” or “inactive”. The state of a modulation depends only on the state of its source: it is “active” if its source is satisfied, and “inactive” otherwise. If the source of the modulation is a unique EPN, it is satisfied if and only if this EPN is “present”; whereas if the source is a logical function structuring some EPNs, it is satisfied if and only if the logical function is satisfied, where an EPN that is “present” counts for true and an EPN that is “absent” for false. As for EPNs and processes, their states may change following the states of the other EPNs and processes of the system: a process may be “switched on” when it can effectively consume its reactants, and “switched off” when it has produced its products; an EPN may become “present” when it can be produced by a process that is “switched on”, and “absent” when it may be consumed by a process. More formally: (iv) a process may be “switched on” if and only if all its reactants are “present”, all the necessary stimulations targeting it are “active”, all the absolute inhibitions targeting it are “inactive”, and at least one stimulation targeting it is “active” or one inhibition targeting it is “inactive”; (v) a process may be “switched off” if and only if all its products are “present”; (vi) an EPN may become “present” if and only if it is the product of a process that is “switched on”; (vii) and finally, an EPN may become “absent” if and only if it is the reactant of a process that is “switched on” and all the products of this process are “present”.

This interpretation is permissive: the constraint to be satisfied for a process to be “switched on” is the weakest possible when still considering the usual meaning of stimulations and inhibitions, and a reactant of a process may never be consumed even if the products of the process have been produced. A model built using this interpretation may be encoded under the form of an AN as follows: (a) each EPN is encoded by one automaton that has two local states “1” and “0”, encoding the “present” and “absent” states of the EPN, respectively; (b) each process is also encoded by one automaton with local states “1” and “0”, encoding this time the “switched on” and “switched off” states of the process, respectively; (c) and transitions between local states of automata are encoded and conditioned straightforwardly from rules (v-vii) described above. For example, an automaton encoding an EPN will have one transition from its state “0” to “1” for each process that produces it, and this transition will be conditioned by the automata modelling the producing process being in its local state “1”, following rule (vi).

The update scheme considered is the asynchronous one, where only one transition may be fired at each time step (in terms of the model, only one process or EPN may change its state at each time step), making the entailed dynamics non-deterministic. An example of an AN-encoded model built from an SBGN PD map under the general semantics is given in Additional file 2: Figure S2.

#### Model analysis

*Construction of the precursor states* The set of precursor states was built based on two constraints: each precursor state (1) should contain the complex formed of the three proteins pRB (unphosphorylated), DP1, and E2F1 and (2) should allow all entities of the map to be reached when not considering effects of modulators on processes. We used Answer Set Programming (ASP) [63] and software *clingo* [64] to compute the set of precursor states. For this, we built an ASP program encoding the reaction graph underlying the merged map (with all modulations removed) and the following rules and constraints:

- *Rule 1*: an entity can be in the precursor state or not;
- *Rule 2*: if an entity is in the precursor state, then it is reachable;
- *Rule 3*: if an entity has a process producing it and consuming an empty set (source), then it is reachable;
- *Rule 4*: if an entity is produced by a process whose reactants are reachable, then it is reachable;
- *Constraint 1*: the precursor state must contain the complex formed of the three proteins pRB (unphosphorylated), DP1 and E2F1;
- *Constraint 2*: all entities must be reachable;
- *Optimization 1*: minimize the total number of entities of the precursor state;
- *Optimization 2*: minimize the total number of state variables set to a value of the entities of the precursor state;
- *Optimization 3*: minimize the total number of complexes of the precursor state.

*Rule 1* generates all possible precursor states. *Rules 2–4* defines reachable entities. *Constraint 1 and 2* constrain the set of output precursor states: an precursor state must contain the aforementioned complex, and all the entities it contains must be reachable. *Optimization 1–3* further reduce the number of output precursor states, by ensuring that they are minimal with respect to cardinality and their number of unmodified and free proteins.

The ASP program was then solved using *clingo*. There were 24 optimal solutions, constituting the 24 precursor states considered in this study.

*Dynamical analysis* We used static analyzer *Pint* [10] to perform the various dynamical analyses of the validation process. For asynchronous automata networks (similarly to asynchronous Boolean networks), the verification of reachability properties has a high computational complexity. However, *Pint* implements a method to reduce the model while preserving the desired reachability property [65]. Reachability properties for phase M of the cell cycle (analysis A and B) were specifically checked using simulation. Deduction of mutations was also realized using *Pint*, which for this relies on static analysis and logic programming techniques which scale to large networks. Such an analysis computes combinations of LoF/GoF mutations making a reachability property false: after applying the mutation, it is guaranteed that no trajectories exist between the specified initial state and a target reachability goal. The analysis returns a list of combinations of mutations, which may be non exhaustive. In our checkpoint analysis, we report each entity of map A which appears in at least one combination of mutations for blocking the reachability of a marker of map B. Finally, we also used *Pint* to extract entities whose activities are necessary in at least one trajectory between a given initial state and a state containing the given markers. This specific computation relies on a causal analysis of transitions, and returns sets of entities such that each trajectory relies on a at least one entity of that set.

## Supplementary Information

Below is the link to the electronic supplementary material.

**Additional file 1.** This file describes modifications of the maps made previous of their merging

**Additional file 2.** This file contains two figures. **Figure S1.** Detailed view of the circadian clock map, visualized in CellDesigner. **Figure S2.** An example of an AN-encoded model built from an SBGN PD map using the general semantics

**Additional file 3.** CellDesigner map of the circadian clock. This map is provided under the CellDesigner format (extended SBML) and can be visualized using CellDesigner

## Data Availability

The circadian clock map introduced in this article is provided in the *CellDesigner* format in the Additional file 3 (CircadianClock.xml). The complete computational analysis, from the merge of the circadian clock and cell cycle maps to the qualitative model validation and mutation predictions, can be repeated using the notebooks and Docker image (based on the CoLoMoTo Docker distribution [12] ) available at https://github.com/pauleve/ClockCycle-notebooks and 10.5281/zenodo.4719400. All analyses were run on a desktop computer with a 3.6Ghz CPU and 256GB of RAM. Total execution time was four hours for all analyses but reachability of phase M (analysis B) which was computed by simulation in less than 24 hours, without parallelization. The merge of the circadian clock and cell cycle maps has been performed with the *csbgnpy* Python library in version 0.1, available at https://github.com/Adrienrougny/csbgnpy. The qualitative interpretation of the coupled map resulting in a formal dynamical model expressed in the autonama network framework has been performed with the *sbgn2an* Python library in version 0.1, available at https://github.com/Adrienrougny/sbgn2an and the *pypint* Python library in version 1.6.1, available at https://loicpauleve.name/pint. The qualitative analysis, including model validation and mutation predictions, has been performed using the software *Pint* in version 2019-05-24, available at https://loicpauleve.name/pint.

## Abbreviations

AN: Automata network
ASP: Answer Set Programming
EPN: Entity Pool Node
GoF: Gain-of-Function
LoF: Loss-of-Function
PD: Process Description
SBGN: Systems Biology Graphical Notation
