# Supplementary material for: A detailed map of coupled circadian clock and cell cycle with qualitative dynamics validation
Source: BMC Bioinformatics. 2021 May 11;22:240. doi: 10.1186/s12859-021-04158-9 (PMC8114686; doi:10.1186/s12859-021-04158-9)
Supplement: Supplementary file 1 — Additional file 1. This file describes modifications of the maps made previous of their merging [file 12859_2021_4158_MOESM1_ESM.pdf]

A detailed map of coupled circadian clock and cell cycle with qualitative dynamics validation
Supplementary information

Adrien Rougny, Loïc Paulevé, Michèle Teboul and Franck Delaunay

1 Modification of the maps for the merging

1.1 Modifications to the cell cycle map

- We added the transcriptional effects of E2F1 (under different forms) on the expression of the cyclin A2, cyclin B1 and cyclin E1 genes that were shown to be necessary for the progression of the mammalian cell cycle [22]. These effects were added under the form of stimulations of processes that produce the proteins from a source rather than on the expression of the genes, because the map did not contain the genes, but did contain the proteins. We considered the following forms for E2F1: in a complex with DP1* only, and in a complex with pRB* phosphorylated one, two or three times.

- We removed the translocation of DP1* from the cytosol to the nucleus and added the translocation its translocation from the nucleus to the cytosol. We also added PP2A as a catalyzer of the dephosphorylation of DP1* in the nucleus.

The following changes were made in the naming in order to ensure that nodes shared with the CLOCK map have a unique identifier, in preparation of the merging:

- We renamed the nucleus compartment to "nucleoplasm" and the cytosol compartment to "cytoplasm"

- We renamed entity pools by replacing the following strings: "p16INK4a*" by "CDKN2A", "p53*" by "TP53", "p21Cip*" by "CDKN1A", "cyclin B1*" by "CCNB1", "CDC2" by "CDK1", "CDK1/CCNB1" by "CCNB1/CDK1".

- We renamed the following state variable: "Ser 1981" to "Ser1981".

- We removed all complexes having both p53 and MDM2 as subunits and all related processes, because they were better described in the CLOCK map.

- We extended the sites of the p53 protein with two undefined and unset state variables because they were present on the p53 protein of the CLOCK map.

- We replaced unphosphorylated WEE1 (at an undefined site) by the more precisely described one of the CLOCK map (having two unphosphorylated sites at Ser53 and Ser472). Analogously we removed WEE1 phosphorylated at an undefined site and added two new forms of WEE1 described in the CLOCK map: phosphorylated at Ser53 and phosphorylated at Ser472. We also added processes producing each form, and catalyzed by phosphorylated CHEK1 for the form phosphorylated at Ser472, and catalyzed by a CDK1/CCNB1 complex for the form phosphorylated at Ser53. Finally, we added the catalysis of the phosphorylation of CDK1 at Tyr15 inside the CDK1/CCNB1 complex by WEE1 phosphorylated at Ser472.

1.2 Modification of the circadian clock map

- CellDesigner does not allow building complex logical functions as inputs of modulations. Hence we replaced the modulations targeting the expression of the Cdkn1a gene by a necessary stimulation having as input a complex logical function programmatically. The necessary stimulation reads as follows: the expression of Cdkn1a is only possible when the Cdkn1a gene is present and either protein TP53 phosphorylated at Ser15 and Ser20 is present or protein RORG is present and protein NR1D1 phosphorylated one time is absent.

- Likewise, we replaced the modulations targeting the expression of the Per2 gene by a necessary stimulation that reads as follows: the expression of Per2 is only possible when the Per2 gene is present and either the complex including GC and acetylated GCR is present, or protein HSF1 is present, or the complex including the CSNK2A protein, phosphorylated and acetylated ARNTL, and protein CLOCK is present and the big complex including protein CSNK1E, protein CRY1, protein CLOCK, protein PER1, phosphorylated and acetylated ATNTL and protein CRY2 is absent.

- Morevoer, CellDesigner does not allow representing absolute inhibitions, while inhibitions of the circadian clock map should be considered as absolute. Hence, we replaced all inhibitions of this map by absolute inhibitions.
