# Supplementary material for: A detailed map of coupled circadian clock and cell cycle with qualitative dynamics validation
Source: BMC Bioinformatics. 2021 May 11;22:240. doi: 10.1186/s12859-021-04158-9 (PMC8114686; doi:10.1186/s12859-021-04158-9)
Supplement: Supplementary file 2 — Additional file 2. This file contains two figures. Figure S1. Detailed view of the circadian clock map, visualized in CellDesigner. Figure S2. An example of an AN-encoded model built from an SBGN PD map using the general semantics [file 12859_2021_4158_MOESM2_ESM.pdf]

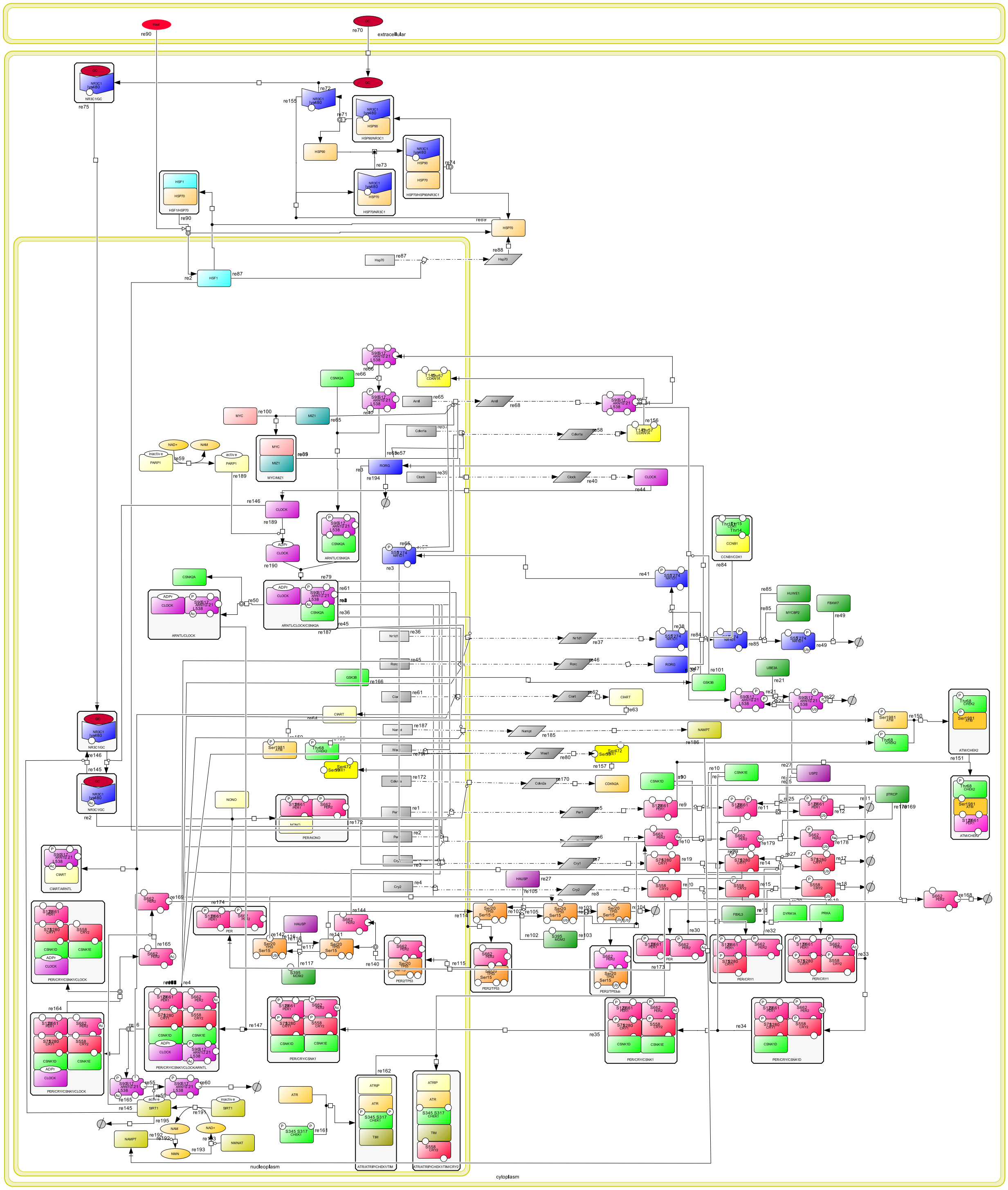

Figure S1: **Detailed view of the circadian clock map, visualized in CellDesigner.** This map represents the molecular interactions underlying the circadian clock. It is represented using the CellDesigner format, which is compatible with the SBGN Process Description language

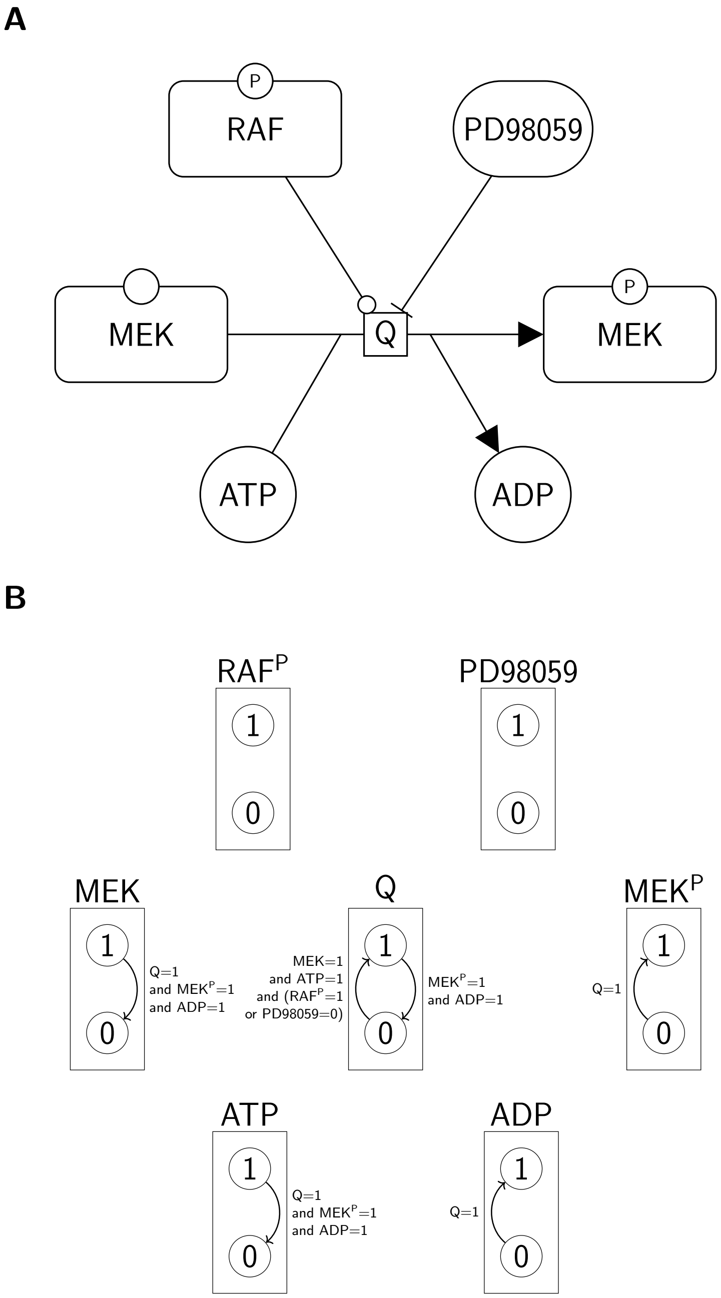

Figure S2: **An AN-encoded model built from an example SBGN PD map under the general semantics.** A. An example SBGN PD map that represents the catalysis of the phosphorylation of MEK by p-RAF and its inhibition by PD98059. B. The AN that encodes the model built from the SBGN PD map under the general semantics. Each rectangle box represents an automaton, and the circles inside the automata represent their local states. Arrows represent transitions between local states, and are conditioned by the states of some automata of the network.
